# Use of Instrumental Physical Therapies and Manual Therapy in Cancer Patients: A Systematic Review

**DOI:** 10.3390/cancers18030385

**Published:** 2026-01-26

**Authors:** Luca Barni, Elio Carrasco Vega, Francesca Nacci, Marco Freddolini, Davide Falchi, Serena Guiducci

**Affiliations:** 1 Independent Researcher, 29071 Malaga, Spain; lucabarnistudio@gmail.com (L.B.); marco.freddolini@gmail.com (M.F.); falcodvd4@gmail.com (D.F.); 2 Department of Physiotherapy, Escuela Universitaria de Osuna, 41640 Seville, Spain; 3 Divisions of Internal Medicine and Rheumatology, Department of Experimental and Clinical Medicine, Azienda Ospedaliero Universitaria Careggi, University of Florence, 50134 Florence, Italy; naccif@aou-careggi.toscana.it (F.N.); serena.guiducci@unifi.it (S.G.)

**Keywords:** cancer, instrumental physical therapy, electrotherapy, laser, shock waves, diathermy, manual therapy, kinesitherapy, physiotherapy, survivor

## Abstract

**Background/Objectives**: The objective of this systematic review was to identify and analyze the available evidence on the indications and contraindications of physical and manual therapies in cancer survivors, including patients with metastatic disease and those with a history of neoplasia in follow-up. **Methods**: A literature search covering the period from 2017 to 2025 was conducted using the following databases: PubMed, NICE, NHS Evidence, PEDro, MEDLINE, and ScienceDirect. Systematic reviews and meta-analyses investigating manual or instrumental physiotherapy interventions in cancer survivors were identified and analyzed according to the PRISMA checklist. **Results**: Of the initial 2232 studies retrieved across the databases, 9 studies met the inclusion criteria. The available evidence suggests a positive effect of physiotherapy interventions on pain reduction in cancer survivors, both during and after chemotherapy. **Conclusions**: The clinical recommendation is to implement instrumental physical therapies and manual therapy as part of a multimodal approach rather than as isolated interventions. Close collaboration with the treating oncologist is recommended to determine whether the tumor type requires treatment to be performed away from the tumor site. No absolute contraindications to physiotherapy were identified, either during or after chemotherapy, including in patients with metastatic disease.

## Simple Summary

Long-term cancer treatment leads to a range of complications, including physical deconditioning and associated physical and psychological consequences, which negatively affect quality of life. Oncology physiotherapy provides valuable support across different phases of the disease; however, to date, no specific guidelines exist regarding the type, timing, or dosage of physiotherapy interventions. The aim of this systematic review was to identify and analyze the available evidence on the indications and contraindications of physical and manual therapies in patients with cancer. Overall, the studies reviewed reported positive effects of both instrumental physical therapies and manual therapy in managing disease-related signs and symptoms. Absolute contraindications were not consistently identified; however, caution is recommended when applying treatments directly over tumor sites, avoiding strong manual pressure, and seeking prior clinical guidance from the treating oncologist.

## 1. Introduction

The World Health Organization defines cancer as “a broad term for a large group of diseases that can affect any part of the body. Other terms used include tumors, malignant tumors, and neoplasms” [1]. Cancer is characterized by uncontrolled cell growth resulting from genetic alterations that disrupt normal cellular function, with metastatic spread representing the leading cause of cancer-related mortality [2].

These genetic alterations may occur during cell division, as a result of DNA damage, or be inherited, and they can arise in any tissue of the body. In many cases, cells proliferate without physiological necessity, fail to undergo programmed cell death, or acquire further genetic damage, leading to the formation of a mass known as a tumor, which may be benign or malignant [3]. Moreover, cancer and its treatments frequently result in impaired quality of life and reduced functional capacity [4,5].

In the long term, cancer treatments are associated with physical deconditioning, altered fatigue perception, metabolic changes—particularly in body composition—and psychological impairments, which may lead to depression and anxiety [6,7].

**Figure 1:**
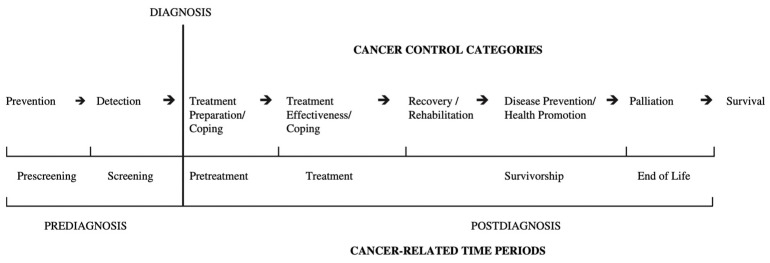
The Physical Activity and Cancer Control Framework [11] (reproduced with approval of the author, Courneya KS et al., 2007).

Oncology physiotherapy provides valuable support across different phases of the disease Figure 1. Physiotherapists operate along a continuum of cancer care, ranging from interventions aimed at counteracting deconditioning and promoting recovery through structured therapeutic exercise to the use of manual and instrumental therapies when clinically indicated [8,9,10].

**Figure 2:**
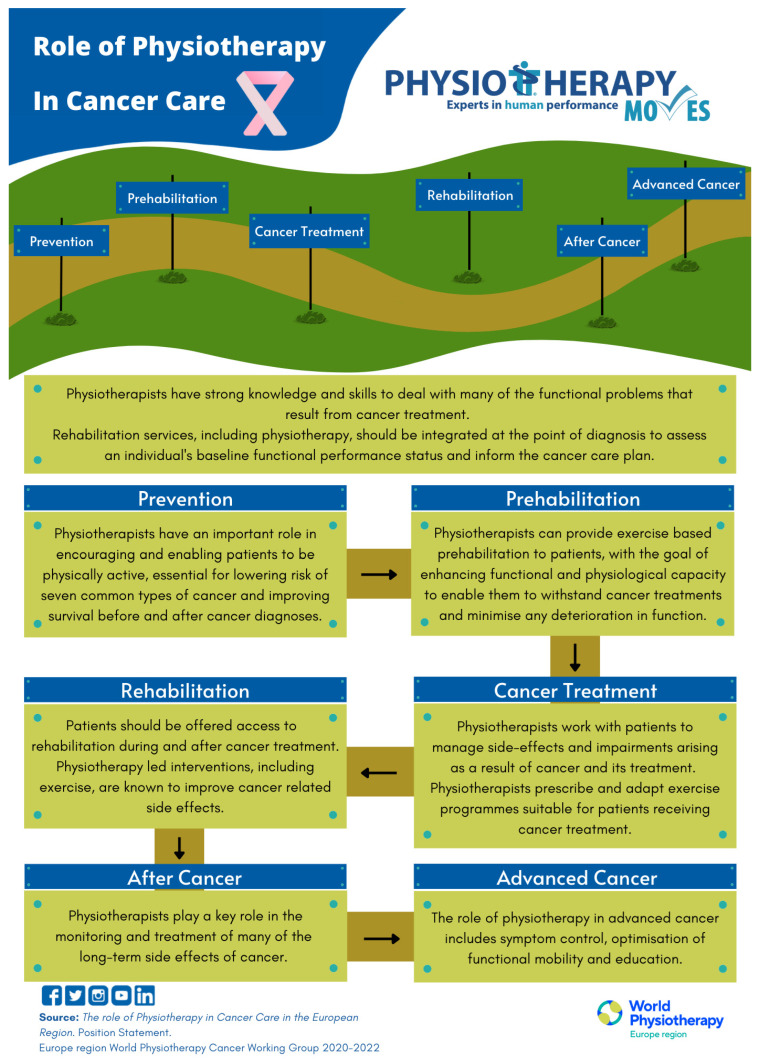
“Role of physiotherapy in cancer care”. Figure kindly provided by Europe Region of World Physiotherapy.

Furthermore, physiotherapists are increasingly integrated as members of the multidisciplinary care team from the time of diagnosis, with the role of assessing patients’ functional performance status, planning individualized rehabilitation interventions, and monitoring health status through regular, scheduled follow-up assessments [8,12] Figure 2.

### Instrumental Physical Therapy and Tumors

Although surgical resection remains the gold standard for the treatment of most solid tumors [13], alternative approaches are available, particularly for patients whose physical or functional condition does not allow for surgery. For this reason, several invasive physical therapies have been developed as ablation methods to destroy tumor cells in situ. These include thermal ablation techniques such as [14]:

- Radiofrequency ablation;
- Microwave ablation;
- Laser ablation.
- Cryoablation.

Radiofrequency ablation (RFA) is one of the most widely used ablation modalities due to the development of devices that are relatively easy to use while maximizing therapeutic efficacy and minimizing associated morbidity [14]. RFA employs low-frequency (460–480 kHz), long-wavelength radio waves to generate heat within the tumor mass, resulting in thermal coagulative necrosis [14,15].

Other forms of instrumental physical therapy utilize acoustic rather than electromagnetic waves. Ultrasound, for example, has been shown to alter tumor and immune cell phenotypes, modulate the tumor microenvironment (TME), enhance beneficial immune cell populations, and facilitate plasmid DNA transfer in sarcoma models. Similarly, shock waves have demonstrated significant cytotoxic effects on tumor cells and tissues, both in vitro and in vivo [16,17].

In clinical physiotherapy practice, instrumental physical therapies such as extracorporeal shock wave therapy, laser therapy, diathermy, and electrotherapy are commonly used to manage musculoskeletal conditions as adjuncts to active exercise, with the aim of reducing pain and restoring function. Moreover, non-invasive physical therapies may help prevent or delay surgical interventions for musculoskeletal disorders [18,19,20].

Manual therapy is a hands-on approach for the treatment of musculoskeletal conditions through mechanical stimulation of muscles, soft tissues, and joints. Techniques such as massage are used to relieve pain, improve flexibility, and promote tissue healing in injured, stiff, or painful areas. Manual or manipulative therapy is primarily performed by osteopaths, chiropractors, and physiotherapists to reduce pain and improve functional outcomes [21].

Several studies have investigated both high- and low-power laser therapy for the management of chemotherapy-related sequelae, such as chemotherapy-induced peripheral neuropathy, as well as for the prevention of acute radiation dermatitis in breast cancer patients. Evidence is also available regarding the use of manual therapy to alleviate pain in patients with various types of cancer. However, due to methodological limitations, these apparently promising results should be interpreted with caution and warrant further systematic analysis and synthesis [22,23,24].

To our knowledge, no systematic review has comprehensively examined the effects of instrumental and manual physical therapies as primary or adjunctive interventions for the management of symptoms and treatment-related side effects in cancer patients or cancer survivors, including their indications and contraindications.

The rationale of this systematic review is to provide clear guidance on the use of manual and instrumental physical therapies in patients with active cancer, as well as in survivors and individuals in follow-up, in order to clarify the safety and efficacy of these interventions and generate clinically relevant knowledge for routine rehabilitation practice.

## 2. Materials and Methods

### 2.1. Study Design

To assess the internal methodological quality of the included studies, the guidelines and recommendations of the Preferred Reporting Items for Systematic Reviews and Meta-Analyses (PRISMA) [25] and the Cochrane Handbook for Systematic Reviews of Interventions [26] were followed. The review protocol was prospectively registered in the PROSPERO database (registration number: CRD420251270101).

### 2.2. Bibliographic Search

A literature search was conducted between June and July 2025 using the following databases: PubMed, NICE, NHS Evidence, PEDro, MEDLINE, and ScienceDirect. Articles published between 2017 and 2025 were considered in order to include the most up-to-date evidence. Eligible publications were limited to articles written in English, French, and Spanish.

The search strategy included the following keywords: “cancer,” “instrumental physical therapy,” “hydrotherapy,” “electrotherapy,” “laser,” “shock waves,” “diathermy,” “manual therapy,” “lymphatic drainage,” “kinesitherapy,” “myofascial release,” “physiotherapy,” and “survivor.” Keywords were combined using the Boolean operators “AND” and “OR” to ensure comprehensive retrieval of relevant studies (e.g., “cancer” AND “physical therapy,” “cancer” AND (“physical therapy” OR “electrotherapy”)). This strategy was applied to all selected physical therapy modalities.

Medical Subject Headings (MeSH) terms used in the search included: “Physical Therapy Modalities,” “Physical Therapy Techniques,” “Neoplasms,” “Electric Stimulation Therapy,” “Lasers,” “Diathermy,” and “High-Energy Shock Waves”.

### 2.3. Study Selection

Study selection was independently performed by two blinded reviewers who screened titles and abstracts according to the predefined inclusion criteria. Following initial screening, discrepancies were discussed and resolved by a third independent reviewer with more than 15 years of experience in conducting systematic reviews. Subsequently, full-text articles were independently assessed by the two reviewers, with any remaining disagreements resolved by the third reviewer.

The inclusion criteria for the study were:

- Systematic reviews or meta-analyses;
- Studies involving individuals affected by cancer or cancer survivors;
- Use of instrumental physical therapies or manual therapies for the management of cancer-related or treatment-related side effects.

The exclusion criteria were:

- Publications in languages other than Spanish, Portuguese, French, English, or Italian;
- Studies involving participants not affected by cancer or not identified as cancer survivors;
- Absence of instrumental or manual physiotherapy interventions;
- Reviews focused on non-physiotherapeutic treatments.

### 2.4. Quality Assessment of the Results and Risk of Bias

The methodological quality of the included studies was independently assessed by three evaluators using the PRISMA 2020 for Abstracts [26]. The PRISMA checklist (File S1) consists of 27 items evaluating the title, abstract, methods, results, discussion, and additional information such as protocol registration, conflicts of interest, and funding sources. Each item was scored as “Yes” (1 point) or “No” (0 points), and total scores were calculated by summing individual item scores.

Study quality was classified into three categories: low quality (<19.0), moderate quality (19.0–22.5), and high quality (>22.5) [26]. The PEDro scale was also used to assess the methodological quality of clinical trials included in the reviewed systematic reviews. This scale evaluates internal validity and interpretability by scoring items on a 0–10 scale. PEDro scores of 0–3 were considered “poor,” 4–5 “fair,” 6–8 “good,” and 9–10 “excellent” [27,28].

## 3. Results

### 3.1. Search and Study Selection

**Figure 3:**
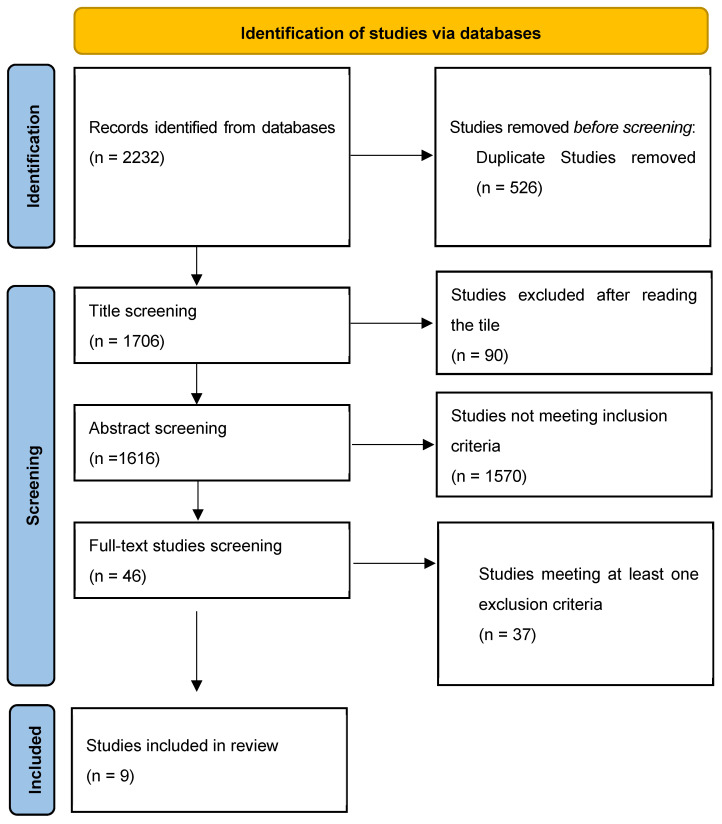
Search flowchart.

The initial database search identified 2232 records. Of these, 526 were excluded as duplicates and 90 were excluded after title screening. A further 1570 articles were excluded for not meeting the main inclusion criteria. Subsequently, 46 full-text articles were assessed for eligibility, of which 37 were excluded for meeting at least one exclusion criterion. Ultimately, 9 studies were included in the review. The study selection process is summarized in the PRISMA flow diagram (Figure 3).

### 3.2. Methodological Quality of Assessment

The qualitative assessment of the 9 included studies [29,30,31,32,33,34,35,36,37] revealed PRISMA checklist scores ranging from 19 to 26. The highest scores were achieved by two studies [33,34], while the lowest scores were reported in two studies [36,37]. The mean PRISMA score across all included studies was 23, indicating overall moderate methodological quality. A summary of the quality assessment is provided in Table 1.

Among the least frequently satisfied PRISMA items were items 13 and 14, corresponding to the summary of methods and the methods used to assess reporting bias, which were not reported in three studies [29,31,37]. Additionally, four studies [29,31,34,37] did not report assessments of the risk of bias due to missing results (item 21). Furthermore, item 8, which concerns the description of methods used to determine eligibility criteria, was not adequately addressed in two studies [34,35].

All studies reported sources of financial and non-financial support as well as potential conflicts of interest. However, in four studies [30,33,35,36], the registration of a study protocol was not reported.

### 3.3. Systematic Review Characteristics

Across the 9 included studies [29,30,31,32,33,34,35,36,37], manual therapy was the most frequently investigated intervention, including techniques such as myofascial induction, myofascial release, classical massage, ischemic compression of trigger points, and myofascial therapy, reported in six studies. Two studies evaluated transcutaneous electrical nerve stimulation (TENS) [30,37], while one study investigated laser therapy, specifically low-level laser therapy (LLLT) [33].

Most systematic reviews synthesized data from randomized controlled trials (RCTs), with the number of included trials ranging from 3 [30] to 13 RCTs [29]. The most commonly used tool for assessing risk of bias was the Cochrane Collaboration’s Risk of Bias tool [38], applied in five of the nine studies [29,32,34,36,37]. The main descriptive characteristics of the included systematic reviews are presented in Table 2.

### 3.4. Systematic Review Results

Overall, manual therapy modalities—such as myofascial induction, myofascial release, acupressure, and massage therapy—were the most frequently reported interventions, with beneficial effects primarily observed in improvements in range of motion and functional outcomes. Two studies reported the use of TENS, demonstrating effects predominantly related to pain management [30,37]. One study evaluated low-level laser therapy (LLLT), reporting primarily anti-edematous effects in the management of lymphedema [33]. Detailed results are presented in Table 3 and Table 4.

## 4. Discussion

The purpose of this systematic review was to analyze and summarize the current evidence regarding the indications and contraindications of instrumental physical and manual therapies in patients with cancer.

Following the analysis of nine studies, evidence emerged supporting a positive effect of physiotherapy interventions on pain relief in cancer survivors, both during and after chemotherapy [29,31,33,35,36,37].

In the six studies that investigated the effects of manual therapies—particularly on pain, range of motion, and quality of life—it was observed that myofascial release techniques, including ischemic compression and classical therapeutic massage, applied either directly to the tumor site (e.g., breast or neck) or indirectly through reflexology on the hands and feet, may contribute to improved quality of life in this patient population. These interventions were associated with reductions in pain, improvements in functional outcomes, and better sleep quality, although results were not always statistically significant [29,31,32,34,35,36]. During stretching and mobilization techniques, maintaining patient comfort throughout the entire treatment session is recommended [35]. Regarding treatment dosage, only one study explicitly reported intervention parameters, suggesting massage durations of 10 to 30 min and treatment programs lasting at least one week [29].

A systematic review and meta-analysis by Chongjie Yao et al. analyzed randomized controlled trials and reported significant reductions in pain intensity associated with manual therapy in certain treatment protocols [24]. Although manual techniques are widely used in clinical practice for patients with cancer, robust quantitative evidence remains limited and heterogeneous, highlighting the need for better-designed randomized controlled trials.

Despite ongoing efforts to improve methodological quality and develop consensus recommendations and practical algorithms for the safe application of physiotherapy interventions in oncology—including exercise, massage, laser therapy, TENS, and balneotherapy [24]—there remains a clear need to reduce heterogeneity in treatment parameters and outcome reporting. This need is particularly evident for manual therapy, as protocols for technologically assisted interventions are typically described in greater detail [39]. Furthermore, additional research is required to more thoroughly investigate the safety profiles of these technologies [40].

With regard to electrotherapy, the two studies analyzed suggest that transcutaneous electrical nerve stimulation (TENS) may represent a safe and user-friendly option that can be self-administered by patients with cancer for pain control. However, TENS is not recommended as a standard treatment for all pain conditions, particularly for chronic chemotherapy-induced peripheral neuropathy [30,37].

The only study evaluating laser therapy—specifically low-level laser therapy (LLLT)—in women treated for breast cancer reported beneficial effects on pain reduction but not on the reduction in limb circumference in cases of lymphedema, a common consequence of surgical and pharmacological treatments [33].

Moreover, across the nine included studies, evidence suggests that in patients with cancer recurrence or metastatic disease, massage therapy—particularly reflexology techniques such as foot reflexology—may be useful to avoid direct manipulation of tumor or metastatic sites while still providing effective pain relief [26,33,41].

Regarding contraindications, no specific or absolute contraindications for the use of instrumental physical or manual therapies were identified in patients with various types of cancer, and no serious or moderate adverse events were reported.

### Clinical Recommendations Based on the Observed Results

Based on the available evidence, instrumental physical and manual therapies should be implemented as part of a multimodal rehabilitation approach rather than as isolated interventions. Direct communication with the treating oncologist is strongly recommended to determine whether the tumor type requires treatment to be performed away from the tumor site. This consideration is particularly relevant for certain malignancies, such as osteosarcoma, where local mechanical stress may be associated with unfavorable prognostic implications, potentially due to prolonged hypoperfusion caused by strong manual pressure. Such hypoperfusion could induce a hypoxic and acidic microenvironment, potentially promoting tumor progression and limiting intratumoral chemotherapy diffusion, thereby reducing treatment efficacy [34,42,43].

In contrast, instrumental physical therapies are less likely to generate high mechanical pressure, which may represent a relative advantage in this patient population. Moreover, electromedical devices commonly used in dermatofunctional physiotherapy or medical oncology typically deliver low-energy stimulation at limited tissue depths, primarily targeting superficial tissues for functional restoration [44].

As previously described, reflex-based approaches that avoid direct treatment of tumor areas—even in patients with metastatic disease—may also be implemented through instrumental physical therapies such as laser therapy and electrotherapy [41,45].

Finally, regarding the biological effects of these modalities, available evidence does not support a cause–effect relationship between the aesthetic use of laser therapy and cancer development [46].

## 5. Conclusions

This systematic review summarizes the available evidence regarding the indications and contraindications of instrumental physical and manual therapies in patients with cancer, with particular emphasis on treatment effectiveness, especially in pain management.

Future research should focus on large, well-designed, multicenter randomized controlled trials with longer follow-up periods in order to provide more comprehensive and robust evidence on the effects of physical and manual therapies in patients with cancer, both during and after oncological treatment.

Overall, the instrumental physical therapies and manual therapies analyzed in the included studies demonstrated positive effects in the management of disease-related signs and symptoms in most cases, without consistently identifying absolute contraindications to treatment. Nevertheless, caution is advised when applying therapies directly over tumor sites, avoiding excessive manual pressure, and ensuring prior clinical consultation with the treating oncologist.

The main limitation of this review lies in the heterogeneity, variability, and overall quality of the study designs, assessment tools, and intervention protocols, which limited direct comparison of outcomes and precluded the performance of meta-analyses. Consequently, further high-quality primary studies are needed to better evaluate treatment effects, define appropriate dosage parameters, and establish standardized methodologies for the safe and effective implementation of dermatofunctional physiotherapy interventions in oncology care.

Clinicians should integrate the currently available clinical evidence to guide the selection of the most appropriate therapeutic approach for each patient and to inform future research directions.

## Figures and Tables

**Table 1:** Scoring of included studies according to the PRISMA scale.

Author, Year	Items																											Total
	1	2	3	4	5	6	7	8	9	10	11	12	13	14	15	16	17	18	19	20	21	22	23	24	25	26	27	
Baxter, 2017 [33]	•	•	•	•	•	•	•	•	•	•	•	•	•	•	•	•	•	•	•	•	•	•	•	-	•	•	•	26
Hsieh, 2021 [34]	•	•	•	•	•	•	•	-	•	•	•	•	•	•	•	•	•	•	•	•	•	•	•	•	•	•	•	26
Hurlow, 2015 [30]	•	•	•	•	•	•	•	•	•	•	•	•	•	-	-	•	•	•	•	•	-	•	•	-	•	•	-	22
Lara-Palomo, 2021 [32]	•	•	•	•	•	•	•	•	-	•	•	•	•	•	•	•	•	•	•	•	•	•	•	•	•	•	•	26
Pattanshetty, 2022 [35]	•	•	•	•	•	•	•	-	•	•	•	•	-	•	•	•	•	•	•	•	•	•	•	-	•	•	-	23
Pinheiro, 2019 [31]	•	•	•	•	•	•	•	•	•	•	•	-	-	-	-	•	•	-	-	•	-	•	•	•	•	•	-	19
Püsküllüoglu, 2022 [37]	•	•	•	•	•	•	•	•	•	•	•	-	-	-	-	•	•	•	-	-	-	-	•	•	•	•	•	19
Samuel, 2021 [36]	•	•	•	•	•	•	•	•	•	•	•	•	•	•	-	•	•	•	•	•	•	•	•	-	•	•	-	24
Zhang, 2023 [29]	•	•	•	•	•	•	•	•	•	•	•	•	-	-	•	•	•	•	-	-	-	-	•	•	•	•	•	21

• = criterion satisfied; - = criterion not satisfied, overall PRISMA score = 23.

**Table 2:** Descriptive characteristics of systematic reviews.

Author, Year	Objectives or Research Question	Search Date	Included Primary Studies	Risk of Bias Assessment Tools
Baxter, 2017 [33]	This systematic review evaluated the effectiveness of LLLT n the management of BCRL.	November 2016	7 RCTs	PEDro scale
Hsieh, 2021 [34]	The aim of this study was to examine the effect of acupressure on fatigue in cancer survivors and the moderators of the effect of acupressure on cancer-related fatigue relief.	July 2020	9 RCTs	Cochrane Collaboration Risk of Bias tool 2.0
Hurlow 2012 [30]	The aim of this systematic review was to determine the effectiveness of TENS for cancer-related pain in adults.	November 2011	3 RCTs	Cochrane risk of bias assessment tool
Lara-Palomo, 2021 [32]	The purpose was to analyze the effectiveness of myofascial therapy on musculoskeletal pain and functionality of the upper extremities in female breast cancer survivors, and to evaluate the changes in range of motion, quality of life, and mood state of these patients.	October 2020	8 RCTs	Cochrane risk of bias assessment tool
Pattanshetty, 2022 [35]	The objective of the present study was to highlight the evidence of the effectiveness of various manual therapy techniques focusing on neck pain and its effect on QOL in HNC survivors that may have clinical relevance.	December 2020	7 RCTs	National Health and Medical Research Council (NHMRC)
Pinheiro da Silva, 2019 [31]	The purpose of this systematic review was to investigate the effectiveness of manual therapy for chronic musculoskeletal pain in the upper limbs and thorax of female breast cancer survivors and to investigate the changes in the quality of life and function of these patients.	March 2018	5 RCTs	PEDro scale
Püsküllüoglu, 2022 [37]	The aim of the current study was to assess whether TENS is effective for pain or chemotherapy-induced peripheral neuropathy management in comparison to sham TENS or no treatment or standard management in adult cancer patients.	May 2020	7 RCTs	Cochrane risk of bias assessment tool
Samuel, 2021 [36]	This systematic review was performed to identify relevant RCTs to determine whether there is currently any evidence to support the use of therapeutic massage or relaxation therapy for improving sleep outcomes in cancer survivors.	September 2020	7 RCTs	Cochrane risk of bias assessment tool
Zhang 2023 [29]	The purpose of this study was to critically evaluate the effect of massage therapy on relieving cancer pain and to provide evidence and reference for the application of massage therapy in clinical cancer nursing.	November 2022	13 RCTs	Cochrane risk of bias assessment tool

Manual therapy (MT); HNC (head and neck cancers); BCRL (breast cancer-related lymphedema); chronic musculoskeletal pain (CMP); LLLT (low-level laser therapy); QOL (quality of life); RCTs (Randomized Controlled Trials); TENS (transcutaneous Electrical Nerve Stimulation).

**Table 3:** Results of manual therapy studies.

Author, Year	Intervention Type	Disease Status	Main Results
Hsieh, 2021 [34]	Acupressure	Cancer survivors	Fourteen articles involving 776 participants with cancers were included. Acupressure considerably alleviated cancer-related general, physical, and mental fatigue (g = −0.87, −0.87, and −0.37) compared with controls. Increasing female percentage of participants significantly reduced the effects of acupressure on fatigue (B = −0.01, *p* < 0.001). The executor and operation approach as well as treatment period during chemotherapy did not moderate the effects of acupressure on fatigue relief.
Lara-Palomo, 2021 [32]	Myofascial therapy	Female breast cancer survivors	A total of eight RCTs were included. The results suggested that myofascial therapy does not have a greater statistically significant immediate effect on pain intensity (SMD: −0.15; 95% CI −0.48, 0.19), functionality (SMD: −0.17; 95% CI −0.43, 0.09) and range of motion in flexion (SMD: 0.30; 95% CI −0.13, 0.74) than an inactive, passive treatment or another intervention. However, a statistically significant result was observed for the abduction shoulder in favor of the experimental group (SMD: 0.46; 95% CI 0.05, 0.87; *p* = 0.03).
Pattanshetty, 2022 [35]	Various manual therapy techniques	HNC survivors	Seven studies were assessed for risk of bias that comprised of three clinical trials, one case series and three case reports that applied Maitland’s mobilisation, myofascial release, and Muscle Energy Techniques to head and neck cancer survivors in various clinical settings. The outcomes highlighted decrease in pain, improvement in cervical range of motion and quality of life.
Pinheiro da Silva, 2019 [31]	Manual therapy	Female breast cancer survivors	The database searches retrieved 1562 titles, and after screening, 5 papers were included for full analysis. The manual therapy techniques described in the included studies involved myofascial induction, myofascial release, classic massage, ischemic compression of trigger points, and myofascial therapy. A meta-analysis, using a fixed-effects model, found that MT decreased CMP intensity (standardized mean difference: 0.32; 95% CI 0.06–0.57), but no significant difference was observed in quality of life after the MT intervention in comparison with a control condition (standardized mean difference: 0.14; 95% CI 0.17–0.46).
Samuel, 2021 [36]	Therapeutic massage or relaxation therapy	Cancer survivors	The search yielded 371 articles, with 4 RCTs studying massage therapy and 3 RCTs studying relaxation therapy included for qualitative analysis. Massage therapy studies showed statistically significant improvements in self-reported sleep questionnaires and objectively recorded long sleep episodes, as assessed via an accelerometer. No significant improvements in sleep outcomes were observed in the relaxation therapy studies, although there were trends for improved self-reported sleep quality.
Zhang, 2023 [29]	Massage therapy	Active cancer or treatment-related pain	Thirteen randomized controlled trials were included in the meta-analysis, containing 1000 patients (498 in the massage therapy group and 502 in the control group). Massage therapy could significantly relieve cancer pain in patients (standardized mean difference = −1.16, 95% confidence interval [−1.39, −0.93], *p* < 0.00001), especially those in the perioperative period and those with hematological malignancies. Foot reflexology and hand acupressure had a moderate effect on cancer pain relief, with hand acupressure being more effective. Massage duration of 10 to 30 min and a program length of ≥1 week had a better effect and could significantly relieve pain. The occurrence of adverse events was reported in 4 of the 13 studies, all of which were no adverse events.

HNC (Head and neck cancers).

**Table 4:** Results of instrumental physical therapy studies.

Author, Year	Intervention Type	Disease Status	Main Results
Baxter, 2017 [33]	LLLT	Breast cancer survivors and lymphedema secondary to breast cancer treatment	LLLT (PBM) is a potential effective treatment for women with BCRL, but the limited published trials highlight the need for high-quality research to determine optimal clinical parameters.
Hurlow, 2012 [30]	TENS	Included active disease and post-treatment chronic pain	Only one additional RCT met the eligibility criteria (24 participants) for this updated review. Although this was a feasibility study, not designed to investigate intervention effect, it suggested that TENS may improve bone pain on movement in a cancer population. The initial review identified two RCTs (64 participants) therefore this review now includes a total of three RCTs (88 participants). These studies were heterogenous with respect to study population, sample size, study design, methodological quality, mode of TENS, treatment duration, method of administration and outcome measures used. In one RCT, there were no significant differences between TENS and placebo in women with chronic pain secondary to breast cancer treatment. In the other RCT, there were no significant differences between acupuncture-type TENS and sham in palliative care patients; this study was underpowered.
Püsküllüoglu, 2022 [37]	TENS	Active disease and post-treatment symptoms	Based on the results, no strict recommendations concerning TENS usage in the cancer patient population could be issued. However, the existing evidence allows us to state that TENS is a safe procedure that may be self-administered by the patients with malignancy in an attempt to relieve different types of pain. There is a need for multi-center, randomized clinical trials with a good methodological design and adequate sample size.

LLLT (low level laser therapy); TENS (transcutaneous Electrical Nerve Stimulation).

## Supplementary Materials

The following supporting information can be downloaded at: https://www.mdpi.com/article/10.3390/cancers18030385/s1, File S1: RISMA 2020 Checklist.
